# Belly Dancer Dyskinesia: A Case Report and Review of Management

**DOI:** 10.7759/cureus.92188

**Published:** 2025-09-12

**Authors:** Ahlam Aboukar, Wadah Ibrahim

**Affiliations:** 1 Department of Neurology, Leicester Royal Infirmary, Leicester, GBR; 2 Department of Pulmonary, Glenfield Hospital, University Hospitals of Leicester National Health Service (NHS) Trust, Leicester, GBR; 3 Department of Respiratory Sciences, University of Leicester, Leicester, GBR

**Keywords:** abdominal myoclonus, belly dancer dyskinesia, benzodiazepines, clonazepam, movement disorder

## Abstract

Belly dancer dyskinesia (BDD) is a rare neurological disorder characterised by repetitive, involuntary contractions of the abdominal muscles. Diagnosis can be challenging, as investigations often fail to reveal an underlying cause. We report the case of an 18-year-old female who presented with sudden-onset, involuntary abdominal contractions. Investigations, including blood tests and magnetic resonance imaging (MRI) of the brain and spine, were unremarkable. Initial treatment with diazepam partially reduced her symptoms, while clonazepam resulted in significant improvement and sustained control. This case highlights the diagnostic challenges of BDD and suggests that clonazepam may be a useful therapeutic option in selected patients.

## Introduction

Belly dancer dyskinesia (BDD) is a rare movement disorder characterised by rhythmic, involuntary contractions of the abdominal wall muscles, producing wave-like abdominal movements [1]. BDD is classified within the spectrum of hyperkinetic movement disorders, manifesting as a rare focal dyskinesia of the abdominal wall [2]. These contractions can be accompanied by abdominal pain and, in most cases, cannot be suppressed or controlled by respiration [1]. The wave-like movement of the abdominal wall resembles the motions of a belly dancer, which is how the condition earned its name. 

The term "belly dancer’s dyskinesia" was first introduced in the literature by Iliceto et al. in 1990, following a study that documented five cases of uncontrollable, writhing abdominal contractions [3]. These contractions were distinct from those seen in axial torsion dystonia or spinal myoclonus, and, in four of the cases, no identifiable cause was found [3]. Since then, further cases have been reported with diverse aetiologies, including drug-induced, trauma-related, functional, or idiopathic origins [4-7].

Diagnosis can be difficult, as standard investigations, including blood tests, neuroimaging, electroencephalograms (EEGs), and electromyograms (EMGs), often fail to identify a cause [1,4]. Management is largely guided by case reports of BDD and similar movement disorders such as spinal myoclonus, with benzodiazepines, antiepileptics, and dopamine antagonists trialled with variable efficacy [1,6,8]. Here, we report a rare case of BDD in a young female with no structural pathology, successfully managed with clonazepam.

## Case presentation

An 18-year-old previously healthy female presented to the emergency department with a three-day history of recurrent involuntary jerking movements of the abdomen. The episodes began suddenly at rest, were repetitive, and were accompanied by hiccups. The contractions were not influenced by respiration, and they caused significant abdominal pain. Over the course of three days, the episodes became more frequent and prolonged, initially lasting minutes but progressing to hours. As the abdominal pain intensified, she experienced a brief loss of consciousness. This lasted approximately one minute, with no associated tongue-biting, urinary incontinence, or limb jerking, and prompted admission to the hospital.

The patient reported recent psychological stress related to university examinations. She denied physical trauma but had been practising new dance choreography involving repetitive backward jumps. She reported no recent medication changes, recreational drug use, or use of herbal remedies. Her regular medications included sertraline and the oral contraceptive co-cyprindiol, which she had been taking for the past two years. She had no history of taking prokinetic, dopaminergic, antiemetic, or antipsychotic medications. She did, however, note that the frequency of abdominal contractions increased after consuming alcohol.

On arrival, she was conscious but agitated, tachycardic, and hypotensive, with transient syncopal episodes. Neurological examination revealed frequent wave-like abdominal wall contractions extending from the costal margin to the umbilicus, which can be seen in Video 1. Cranial nerves, reflexes, and muscle tone were intact, with no fasciculations or limb involvement. 

**Video 1:** Patient in the supine position demonstrating involuntary, rapid, repetitive movements of the anterior abdominal wall.

**Figure 1:**
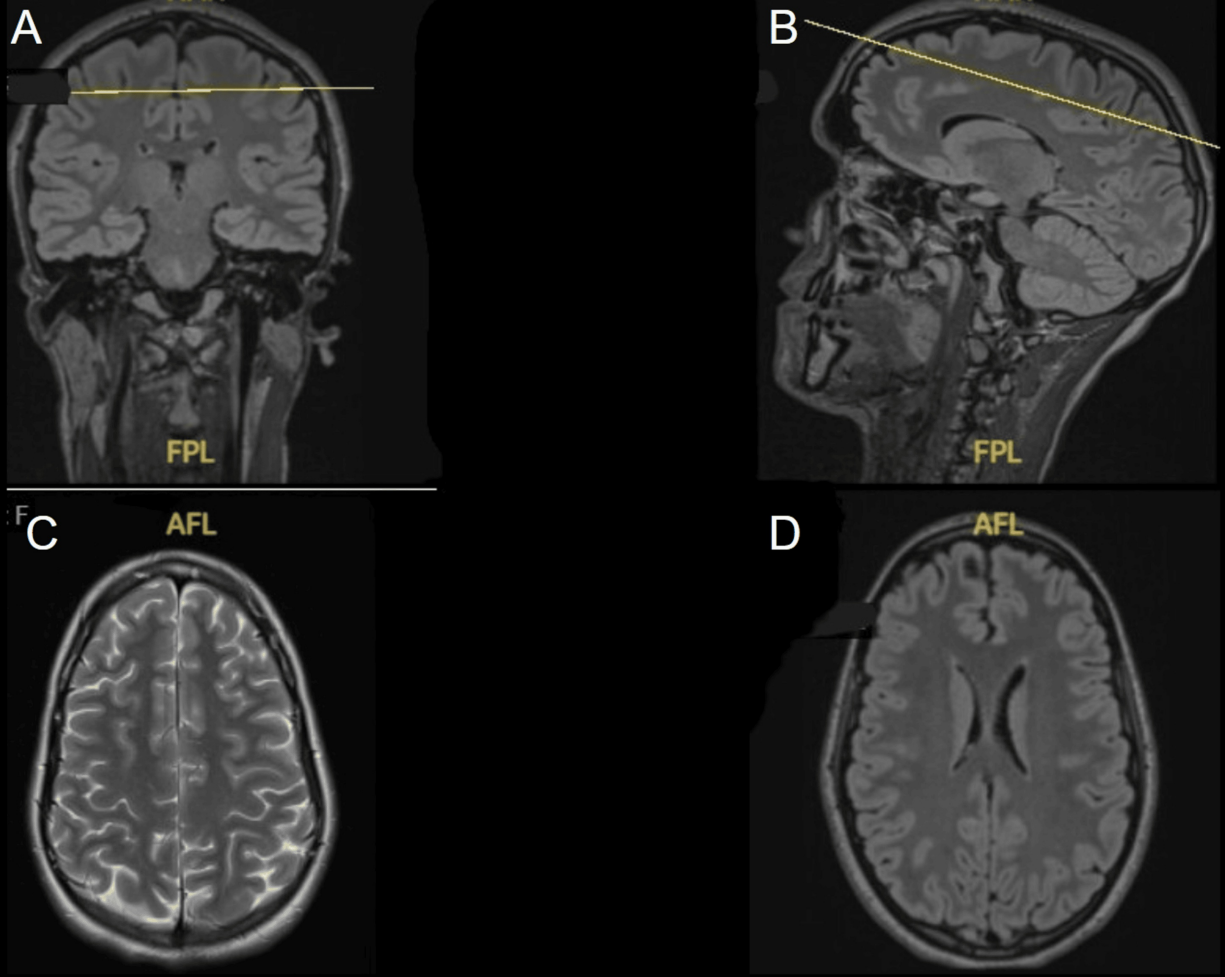
MRI of the brain (A–D) demonstrating normal sagittal, coronal, and axial views. No evidence of focal lesion. No findings to suggest inflammatory/demyelinating pathology.

**Figure 2:**
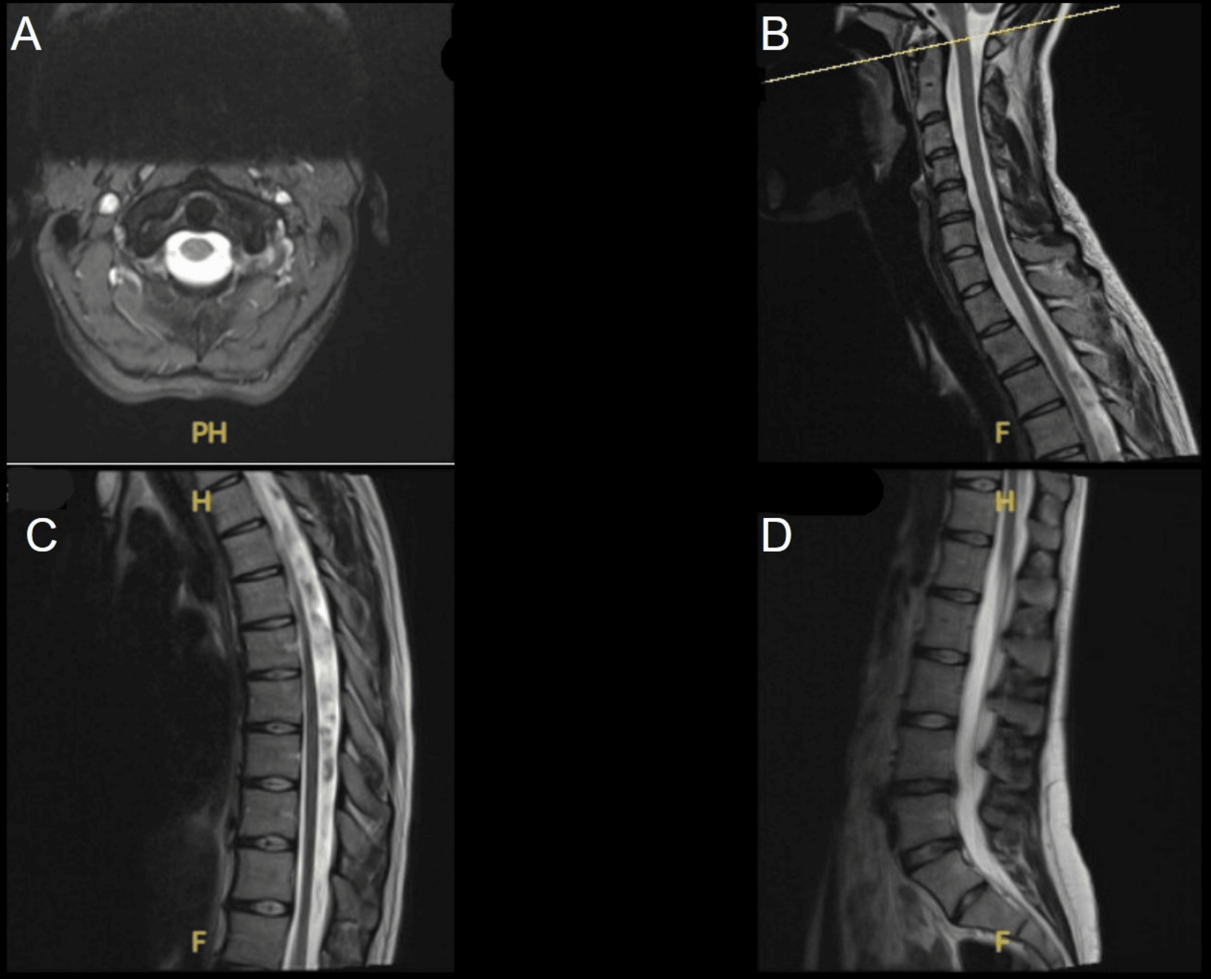
MRI of the spine (A–D) showing the cervical, thoracic and lumbar regions. No evidence of focal lesion. No findings to suggest inflammatory/demyelinating pathology.

Routine blood tests, including electrolytes, thyroid function, and inflammatory and infection markers, were normal. The chest radiograph was also unremarkable. MRI of the brain and spine revealed no abnormalities, excluding demyelination, inflammation, or focal lesions (Figures 1, 2). 

The patient was initially treated with diazepam 5 mg, which partially reduced the contraction frequency. Clonazepam was introduced under neurology guidance at 0.5 mg at night, which was subsequently increased to 1 mg twice a day. This resulted in a significant reduction in her symptoms; however, the patient reported extreme fatigue when increasing the dose. At discharge, her symptoms were well controlled with clonazepam 0.25 mg as required.

Two months later, the patient was followed up, and she reported sustained improvement, with only one further episode of contractions, effectively managed with clonazepam 0.25 mg. Since her clinic follow-up, she reports complete resolution of her symptoms with no further episodes. She has been able to continue her studies and usual activities with no residual effects.

## Discussion

Belly dancer dyskinesia is characterised by involuntary contractions of abdominal muscles, often resembling belly dancing movements [1,3]. It can be differentiated from diaphragmatic myoclonus, propriospinal myoclonus, truncal dystonia, and functional movement disorders, as the abdominal wall contractions are not linked to respiration, lack dystonic posturing, and persist despite distraction [9]. Aetiologies reported include drug-induced cases linked to clebopride and levodopa [4,5], trauma-related presentations such as cervical root injury [6], and functional or stress-related disorders [7]. Notably in this case, alcohol appeared to exacerbate symptoms, an observation that has not been widely described in previous literature. 

Given the wide range of potential causes, a systematic approach is required to ascertain the underlying aetiology. A comprehensive history should focus on recent or chronic medication use, exposure to dopamine agonists/antagonists, illicit substances, prior surgery, traumas, pregnancy, or significant psychological stressors. Physical and neurological examinations are essential to exclude signs of spinal or peripheral nerve involvement, dystonia, or functional features such as distractibility or variability of symptoms. 

Investigations should include baseline laboratory studies (electrolytes, thyroid function, inflammatory markers, vitamin B₁₂), and targeted imaging such as MRI of the brain and spine to exclude demyelination, inflammation, or structural lesions. Electrophysiological studies, including EMG, may help characterise rhythmic discharges, while EEG can exclude epileptic correlates. These were not indicated in our case, as the patient was asymptomatic at follow-up. In uncertain cases, neurophysiological testing such as Bereitschaftspotential recording, a measure of activity in the motor cortex and supplementary motor area of the brain leading up to voluntary muscle movement, may assist in differentiating functional from organic causes [7,10]. This structured approach can help clinicians narrow the differential diagnosis and guide appropriate management. 

Pharmacological management remains empirical. Antiepileptics such as valproate and carbamazepine have been trialled in suspected spinal segmental myoclonus with variable responses [8]. Benzodiazepines (clonazepam, diazepam) have also been trialled, with partial or complete relief attributed to GABAergic inhibitory mechanisms [1,11]. Diazepam has a rapid onset with peak effect within 1-1.5 hours, while clonazepam reaches peak levels after 1-4 hours [12,13]. This may explain why the patient in this case experienced sustained symptom relief with clonazepam. Chlorpromazine, a dopamine antagonist, has been shown to be effective in a post-traumatic case of BDD [6]. Baclofen has also been used, but with inconsistent outcomes [11]. In refractory cases of BDD, botulinum toxin A injections and phrenic nerve blocks have provided symptomatic relief [14]. No single therapy has proven universally effective, underscoring the need for an individualised approach based on clinical context and potential aetiology.

## Conclusions

Belly dancer dyskinesia is a rare and diagnostically challenging condition with diverse potential aetiologies. A systematic assessment, including detailed history, examination, and appropriate investigations, is essential to exclude structural, drug-induced, or functional causes. While no standardised treatment exists, clonazepam has been shown to be effective for this patient. This case highlights the importance of recognising BDD as a differential for unexplained abdominal movements and emphasises the need for an individualised, patient-centred approach to management. However, further case reports and literature are required to help establish optimal diagnostic strategies and treatment pathways. 
